# Impairment of Type I but Not Type III IFN Signaling by Hepatitis C Virus Infection Influences Antiviral Responses in Primary Human Hepatocytes

**DOI:** 10.1371/journal.pone.0121734

**Published:** 2015-03-31

**Authors:** Jacques Friborg, Petra Ross-Macdonald, Jian Cao, Ryan Willard, Baiqing Lin, Betsy Eggers, Fiona McPhee

**Affiliations:** 1 Discovery Virology, Bristol-Myers Squibb Research and Development, Wallingford, Connecticut, United States of America, 06492; 2 Applied Genomics, Bristol-Myers Squibb Research and Development, Hopewell, NJ, United States of America; Temple University School of Medicine, UNITED STATES

## Abstract

Peginterferon lambda-1a (Lambda), a type III interferon (IFN), acts through a unique receptor complex with limited cellular expression outside the liver which may result in a differentiated tolerability profile compared to peginterferon alfa (alfa). In Phase 2b clinical studies, Lambda administered in combination with ribavirin (RBV) was efficacious in patients with hepatitis C virus (HCV) infection representing genotypes 1 through 4, and was associated with more rapid declines in HCV RNA compared to alfa plus RBV. To gain insights into potential mechanisms for this finding, we investigated the effects of HCV replication on IFN signaling in primary human hepatocytes (PHH) and in induced hepatocyte-like cells (iHLCs). HCV infection resulted in rapid down-regulation of the type I IFN-α receptor subunit 1 (IFNAR1) transcript in hepatocytes while the transcriptional level of the unique IFN-λ receptor subunit IL28RA was transiently increased. In line with this observation, IFN signaling was selectively impaired in infected cells upon stimulation with alfa but not in response to Lambda. Importantly, in contrast to alfa, Lambda was able to induce IFN-stimulated gene (ISG) expression in HCV-infected hepatocytes, reflecting the onset of innate responses. Moreover, global transcriptome analysis in hepatocytes indicated that Lambda stimulation prolonged the expression of various ISGs that are potentially beneficial to antiviral defense mechanisms. Collectively, these observed effects of HCV infection on IFN receptor expression and signaling within infected hepatocytes provide a possible explanation for the more pronounced early virologic responses observed in patients treated with Lambda compared to alfa.

## Introduction

For the last decade, the combination of peginterferon alfa (alfa) and ribavirin (RBV) has been the backbone of therapy for chronic Hepatitis C virus (HCV) infection. Successful treatment, defined as sustained virologic response (SVR) following completion of therapy, is achieved in 40 to 50% of patients infected with HCV genotype (GT)-1 (-1a and -1b) and 78 to 86% of those infected with HCV GT-2 or -3.[1] In recent years, highly selective direct acting antivirals (DAAs) targeting the HCV non-structural 3/4A (NS3/4A) protease or the NS5B polymerase have become available marking a new era in the management of chronic HCV infection. Use of these agents in combination with alfa plus RBV results in improved SVR rates (66 to 89%) in treatment-naive patients infected with HCV GT-1.[2–5] In contrast, the virologic response to these treatments is lower among patient populations who previously failed alfa plus RBV therapy.[6, 7] Host genetic variations and viral determinants have been shown to influence responsiveness to alfa treatment.[8] Nonetheless, the molecular mechanisms modulating sensitivity to alfa in HCV patients have yet to be defined and continue to pose a substantial hurdle in the design of tailored interferon (IFN)-based regimens.

A hallmark of the innate immune response against viral infection is the rapid production of type I (IFN-α and IFN-β) and type III IFNs (IFN-λ-1, -2 and -3, also known as IL-29, IL-28A and IL-28B, respectively) triggered by a network of pattern recognition receptors (PRRs) expressed in the host cell. These sensors can recognize a variety of pathogen-associated molecular patterns (PAMPs) within viral products. During HCV infection, IFNs activate the Jak (*Janus* kinase)-STAT (signal transducer and activator of transcription) pathway to create an antiviral state, both within infected hepatocytes and in neighboring uninfected cells, through the expression and complementary action of numerous IFN-stimulated genes (ISGs).[9, 10] The release of IFNs also leads to the recruitment and activation of immune effector cells, through transcription of pro-inflammatory cytokines and chemokines, thereby linking the innate and adaptive arms of immunity crucial to the host ability to control acute HCV infection.[11] Despite the potential effectiveness of these immune defenses, spontaneous clearance of HCV is only observed in 20–30% of infected individuals while the majority of patients progress to chronic infection. This disease progression is partially explained by the fact that HCV has evolved various strategies over time to disrupt the host hepatic innate response and evade antiviral defenses.[12] Moreover, it has now been established that the status of the endogenous hepatic IFN system is a strong predictor of responsiveness to alfa. Several transcriptional studies conducted on liver biopsy specimens from patients with chronic HCV infection have shown that pre-activated baseline ISG patterns correlate with a poor response to an alfa plus RBV regimen.[13–15] There is compelling evidence suggesting that the innate response induced during HCV infection may lead to a refractory state in hepatocytes to further IFN-α stimulation. The activation of IFN-α is tightly regulated by several negative feedback loops mediated by cellular factors such as suppressors of cytokine signaling (SOCS) family members and the ubiquitin-specific peptidase 18 (USP18/UBP43).[16–18] The significant induction of ISGs and desensitization of hepatocytes to IFN-α may in part explain non-responsiveness to alfa-based treatment in patients.

Recent studies suggest that type III IFNs play an important role in the hepatic innate response against HCV infection. In cell culture systems, primary human hepatocytes have the ability to produce (predominantly) type III IFNs in response to HCV infection.[19] The level of IFN-λ-1 produced *in vitro* was shown to be sufficient for abrogating viral replication in primary human hepatocytes, and was therefore proposed to limit growth and spread of HCV infection within liver tissues. In Chimpanzees, HCV infection resulted in limited expression of type I IFNs but rapid induction of IFN-λ-3 associated with an up-regulation of ISGs in liver biopsies.[20] Moreover, several landmark genome-wide association studies (GWAS) have independently identified single nucleotide polymorphisms upstream of the *IL28B* locus (IFN-λ-3 gene) as strong predictors of an early spontaneous viral clearance and a more favorable outcome to alfa-based therapies in patients with GT-1 HCV infection.[21] The link between HCV clearance and the *IL28B* genotype highlights the potential importance of type III IFN responses in patients refractory to alfa.

Results from Phase 2b trials investigating the efficacy and safety of a combined regimen of Lambda plus RBV in chronically infected HCV patients with GT-1 to GT-4 were recently reported.[22–24] Interim analysis indicated that Lambda regimen led to significantly more rapid declines in viral load at Week 4 (all GTs) and Week 12 (GT-1, -4) on-treatment compared to alfa. By Week 24 of treatment, however, viral suppression was similar in patients receiving the alfa regimen versus the Lambda regimen. Therefore, SVR rates at Week 24 post-treatment (SVR_24_) with Lambda were comparable to those with alfa for GT-1, -4 (37–46% Lambda; 37% alfa) and GT-2, -3 (60%-76% Lambda; 53% alfa). Since type I and type III IFNs are purported to act via the same signaling pathway, understanding the basis of such differential early virological responses to Lambda as compared to alfa may offer additional insights in the mechanisms of IFN responsiveness.

It has been shown that patients with a rapid virological response as defined by undetectable HCV RNA at Week 4 on-treatment are more likely to achieve SVR.[25, 26] Several mathematical models have indicated that HCV RNA levels decline in a biphasic manner following initiation of alfa-based therapy in chronically infected patients.[27–29] The first-phase of decline is rapid, dose-dependent, and usually occurs within 48 h after initiating treatment. Subsequently, a slower second-phase of viral decay ensues which is independent of alfa dosing, and with rates highly variable between patients. The slope of the first-phase can be attributed to the blockade of viral production and/or clearance of free virions in the liver, whereas a second-phase slope appears to reflect the loss of HCV-infected cells. Therefore, a robust biphasic HCV RNA decline is essential for optimal outcome to alfa-based therapy. In line with this concept, a multivariable linear regression analysis has associated the early more rapid virological responses to Lambda plus RBV at Weeks 4 and 12 of treatment, with a faster first-phase HCV RNA decline compared to alfa.[30] Although a complete mechanistic understanding of this observation remains elusive, these fundamental differences may suggest early disparate intrahepatic antiviral responses to treatment with either agent. In this study, we provide evidence that Lambda and alfa trigger distinct antiviral defenses in HCV-infected hepatocytes.

## Materials and Methods

### Primary human hepatocytes and induced hepatocyte-like cells

Primary human hepatocytes (PHH) from healthy donors (Invitrogen) were seeded in collagen coated 12-well plates (Invitrogen) at 8 x 10^5^ cells per well and maintained with daily replacement in Williams E medium containing cell maintenance supplement reagents (Invitrogen) according to the manufacturer’s protocol. Induced hepatocyte like-cells (iHLCs), commercially obtained (Cellular Dynamics), were seeded at 7.6 x 10^5^ or 6.4 x 10^4^ cells per well in collagen coated 12-well plates (Invitrogen) or 96-well plates (Becton Dickinson), respectively. After overnight plating, iHLCs were propagated in maintenance medium containing B27 supplement, oncostatin M and dexamethasone, according to the manufacturer’s protocol. Cultures were maintained for >12 days with medium replacement every 2 days or less, depending on the experiment. Differentiated iHLCs were shown to express hepatic markers and known host factors required for HCV attachment (CD81, SR-B1, occludin, and claudin-1) at levels consistent with those of PHH.

### Compounds

Pegylated lambda-1a (Lambda) is a covalent conjugate form of recombinant (r) IFN-λ-1 and a 20 kDa linear polyethylene glycol chain synthesized at ZymoGenetics, Inc (Seattle, WA). The pegylated form of IFN-α-2a (PEGASYS^®^; alfa) was purchased from Hoffman-La Roche, Inc. (Nutley, NJ). The monoclonal antibody directed against the extracellular domain of the IL28RA receptor subunit (clonal hybridoma E10889) was synthesized at ZymoGenetics, Inc. The NS3 protease inhibitor (PI) asunaprevir (ASV), synthesized by Bristol-Myers Squibb Co., has been previously described.[31]

### Viruses

The infectious HCV cell-culture adapted (HCVcc) GT-2a chimeric virus (J6/JFH-1) has been previously described.[32] After electroporation of *in vitro* transcribed J6/JFH-1 RNA in the Huh-7.5 hepatoma cell line [33], cells were plated for overnight incubation (37°C, 5% CO_2_) and subsequently maintained in culture with daily media replacement. On Day 3, 4 and 5, culture supernatants were harvested using low-speed centrifugation and cleared through 0.45 μm filter units (Millipore Corporation). Filtrates were used to generate viral stocks following inoculation of naive Huh-7.5 cells. Titers of infectious HCVcc in culture supernatants were determined by intracellular immunostaining of the virally-encoded core protein as previously described [33] using a monoclonal anti-core antibody (Affinity Bioreagents). Aliquots of HCVcc were placed in six-well plates and exposed to 120 mJoule/cm^2^ of ultraviolet (UV) light in a Stratalinker 2400 (Statagene). UV-irradiated HCVcc aliquots were confirmed as being non-infectious, as determined by inoculation on naive Huh-7.5 cells. To demonstrate HCVcc infection, antiviral activities of various classes of HCV inhibitors were confirmed following inoculation of naive Huh7-5 cells, PHHs or iHLCs with infectious supernatants. Patient serum with HCV (HCVser) representing GT-1b (2.3 x 10^6^ IU/mL) and GT-3a (10.7 x 10^6^ IU/mL) were commercially obtained (Boca Biolistics, LLC). All infections with either HCVcc or HCVser were performed by incubation of viral inoculums with cells for 6–8 hours (37°C, 5% CO_2_). Cell cultures were subsequently washed twice with Dulbecco’s phosphate buffered saline (D-PBS) and further propagated in maintenance medium for the indicated time points. Naive cells were mock-infected in parallel with maintenance medium free of viruses and served as control. For all experiments, Inhibition of viral infection was confirmed using various classes of HCV inhibitors.

### Microarray analysis

For global transcriptome analysis, total RNA was isolated from PHH (three independent biological replicates) or IHLCs at the indicated time points using the RNeasy Mini Kit (Qiagen). RNA integrity was confirmed on a Bioanalyzer 2100 (Agilent Technologies) prior to labeling using 3’-IVT Express (Affymetrix). Microarray analysis was performed on the Human Genome U219 Array Plate using the Gene Titan MC instrument (Affymetrix). The cel files were processed using the robust multi-array analysis (RMA) algorithm.[34] Probe sets on the HG-U219 array were mapped to loci using the criterion of >80% sequence identity to the loci annotated in RefSeq release 39.[35] Statistical analysis and visualization of expression profiling data was performed in Partek Genomic Suite v6.6. Reported P values are from contrasts specified in a one-way ANOVA on RMA values using Method of Moments. Microarray data are available in the ArrayExpress database (www.ebi.ac.uk/arrayexpress) under accession numbers E-MTAB-2476 and -2477.

### Western immunoblot analysis

To monitor the capacity of Lambda and alfa to induce Jak-STAT signaling in HCV-infected cells, phosphorylation of STAT1 was monitored after stimulation with each respective IFN. Briefly, iHLCs were infected with HCVcc at multiplicity of infection (MOI) of 0.2 and incubated for 4–6 days in presence or absence of a neutralizing monoclonal antibody directed against the extracellular domain of the IL28RA receptor subunit (ZymoGenetics). Mock- (naive) and HCVcc-infected iHLCs were treated at the indicated concentrations with Lambda or alfa for 15 minutes. Cells were then washed once with D-PBS and lysed with RIPA buffer (SIGMA) supplemented with protease inhibitors and phosphatase inhibitors cocktails (SIGMA). Equal amounts of protein lysates were subjected to immunoblotting using antibodies directed against phospho-STAT1 (Tyr701) or STAT1 (Cell Signaling), as previously described.[36] To assess IFN receptor expression, naive and HCV-infected iHLCs were harvested at the indicated time points and subjected to immunoblotting. Primary antibodies to IFNAR1, IFNAR2 and IL28RA were purchased from Lifespan Biosciences (Seattle, WA). IL10RB and β-actin antibodies were purchased from R&D Systems (Minneapolis, MN) and Cell Signaling (Beverly, MA), respectively. Proteins were visualized using ImageQuant technology (GE Healthcare Life Sciences). Densitometry analysis of specific bands was quantified with ImageQuant TL software (GE Healthcare), normalized to β-actin levels in each sample, and expressed as percentage relative to mock-infected cells.

### Luminex bead assay

Following HCVcc infection in 96-well black-clear assay plates, iHLCs received 10-fold serial dilutions of test compounds and were incubated for 30 minutes. Following incubation, media was removed and cells were washed with ice-cold D-PBS to stop the reaction. Subsequently, 50 μL Milliplex MAP lysis buffer (Millipore Corporation) was added to each well, and then agitated for 20 minutes at 2–8°C. Supernatants were collected and status of STAT1 molecules in the samples was monitored by Luminex technology using the Milliplex Phospho-STAT1 assay (Millipore Corporation). Median Fluorescence Intensity (MFI) values for each test compound concentration were exported for subsequent analysis.

### Fluorescence microscopy analysis

Mock- (naive) or HCVcc-infected iHLCs were fixed with 4% paraformaldehyde and permeabilized prior to antibody staining. Cells were then incubated overnight at room temperature with antibodies to the HCV core protein (Affinity Bioreagents), IFNAR1 (Lifespan Biosciences), phospho-STAT1 (Tyr701) or Mx1 (Thermo Scientific). Primary antibodies were subsequently incubated for 1 h at room temperature with Alexa Fluor^488^- and Alexa Fluor^594^-coupled secondary antibodies (Molecular Probes) as recommended by the manufacturer’s instructions. Nuclei were counterstained with Hoechst dye and cells were visualized under a Nikon Eclipse TE 300 microscope. Images were processed with Q-Capture PRO 7 software (QImaging).

### Reverse transcriptase polymerase chain reaction (RT-PCR) analysis

Intracellular HCV RNA quantification was performed using real-time quantitative RT-PCR. Briefly, total RNA was isolated from naive and HCV-infected iHLCs using RNeasy mini kit (Qiagen) and cDNA synthesis was performed using OneStep RT-qPCR kit (Invitrogen) accordingly to manufacturer’s instructions. Amplifications from the conserved HCV 5’ non-coding region were conducted using the following primers and dual-labeled 6-carboxyfluorescein (6FAM)—black hole quencher-1 (BHQ1) probe (Sigma Aldrich): 5’-CCCTGTGAGGAACTACTGTCTT-3’; 5’-GCTGCACGACACTCATACTAAC-3’; and 5’-6FAM-CGCAGAAAGCGTCTAGCCATGG-BHQ1-3’. HCV RNA copy numbers were determined using standard curve generated with 10-fold dilution of RNA transcribed from the full-length J6/JFH1 plasmid. To analyze expression of IFNAR1 transcripts, amplification of cDNA was performed using the following primers and TaqMan minor groove binding (MGB) probe (Applied Biosystems); 5’-CACCATTTCGCAAAGCTCAGA-3’; 5’-TCACTATTGCCTTATCTTCAGCTTCTA-3’; and 6FAM-TGGTCCTCCAGAAGTA-BHQ1-3’. Relative amounts of mRNA were normalized to cellular glyceraldehyde-3-phospate dehydrogenase (GAPDH) levels in each sample. Relative changes in mRNA expression levels were determined using the delta 2^-ΔΔ CT^ method.[37]

### Statistical analysis

GraphPad Prism version 5.01 (GraphPad Software, Inc) was used for statistical analyses as indicated. Differences with *P* < 0.05 were considered statistically significant.

## Results

### HCV infection down-regulates the IFNAR1 transcript

**Fig 1:**
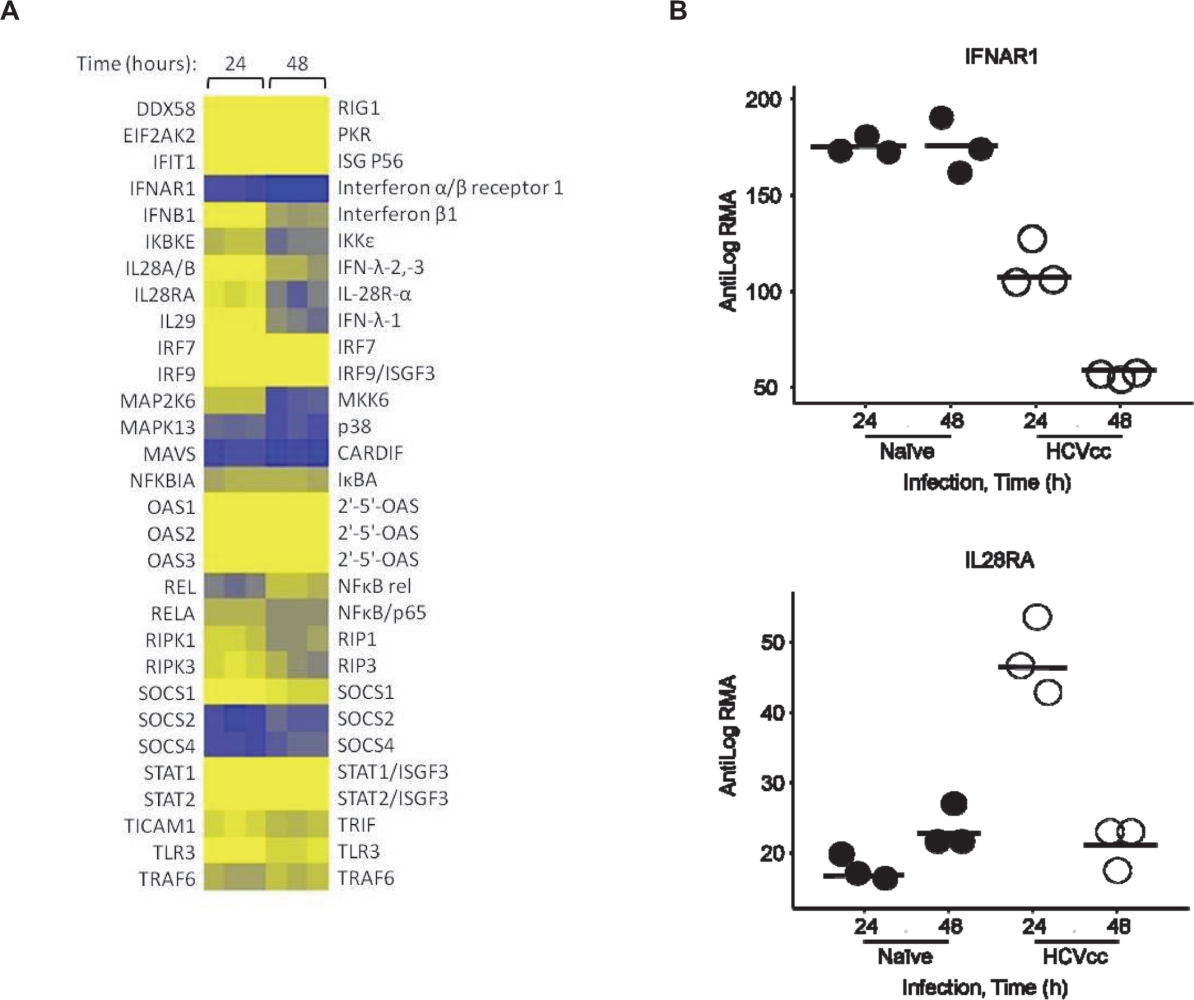
HCV infection induces transcriptional down-regulation of IFNAR1 in PHH. Transcriptional profiling of PHH infected with HCVcc at an MOI of 0.2. (A) Relative expression of genes involved in the PAMP recognition pathway showing data from three replicates at 24 h or 48 h post-infection normalized to naive PHH at the same time points. HUGO nomenclature for the genes is to the left. Statistical analysis was performed using one-way ANOVA and *P* values reported in S1 Table (B) Expression levels of IFNAR1 and IL28RA transcripts in naive (closed circle) and HCVcc-infected (open circle) PHH. The data points represent the RMA values from each individual analyte and horizontal bars indicate the mean levels from three biological replicates. The data shown is representative of two independent experiments.

To gain insight into the effects of HCV on IFN signaling, a transcriptional profiling analysis was performed in PHH following infection with HCVcc. Examination of 76 components of the PAMP recognition pathway identified 30 genes significantly modulated by HCVcc infection at one or both time points using a cutoff of *P* < 0.01 and fold changes greater than 1.5 (S1 Table). Approximately 70% of these genes were up-regulated by 24 h post-infection, but for the most part returned to baseline at the later time point (Fig. 1A). Innate sensing of HCV in hepatocytes occurs through the retinoic acid inducible gene-I (RIG-I) pathway before the onset of extensive viral protein synthesis, whereas toll-like receptor 3 (TLR3) functions as a secondary immune surveillance system by detecting viral RNA intermediates exposed either in endosomes or autophagic vesicles.[12] In agreement with this model, constitutively expressed RIG-I components, such as CARDIF and IKKε, were down-regulated at 24 h or at 48 h post-infection (Fig. 1A), respectively. Conversely, a number of adaptor genes involved in TLR3 signaling such as TRIF, TRAF6 and RIP1 were up-regulated within 24–48 hours of infection. Noteworthy, regulators of IFN-α signaling, SOCS1 and USP18, were also up-regulated in HCV-infected PHH (S1 Table). As previously reported [19, 20], transcriptional induction of the type III IFNs IL-29 (12.5-fold) and IL-28A/B (2.7-fold), and also the type I IFN-β1 (5.4-fold) was observed in infected cells. In contrast, expression of 18 components of the PAMP recognition pathway, including IFN-α family members, IFN-β2 and IFN-γ, was unaffected by HCVcc. Interestingly, infection resulted in the inverse modulation of type I and type III IFN primary co-receptor transcripts (Fig. 1B). Transcript levels of the IFNAR1 subunit progressively decreased during the course of HCVcc infection (48 h: *P* = 1.4 x 10^–7^; S1 Table), whereas the IL28RA transcript was transiently up-regulated at 24 h (*P* = 1.4 x 10^–7^; S1 Table). Transcriptional levels of type I and type III co-receptors, IFNAR2 and IL10RB, respectively, were not affected in our analysis (data not shown).

### Persistent HCV replication alters the expression of IFNAR1 but not IL28RA

**Fig 2:**
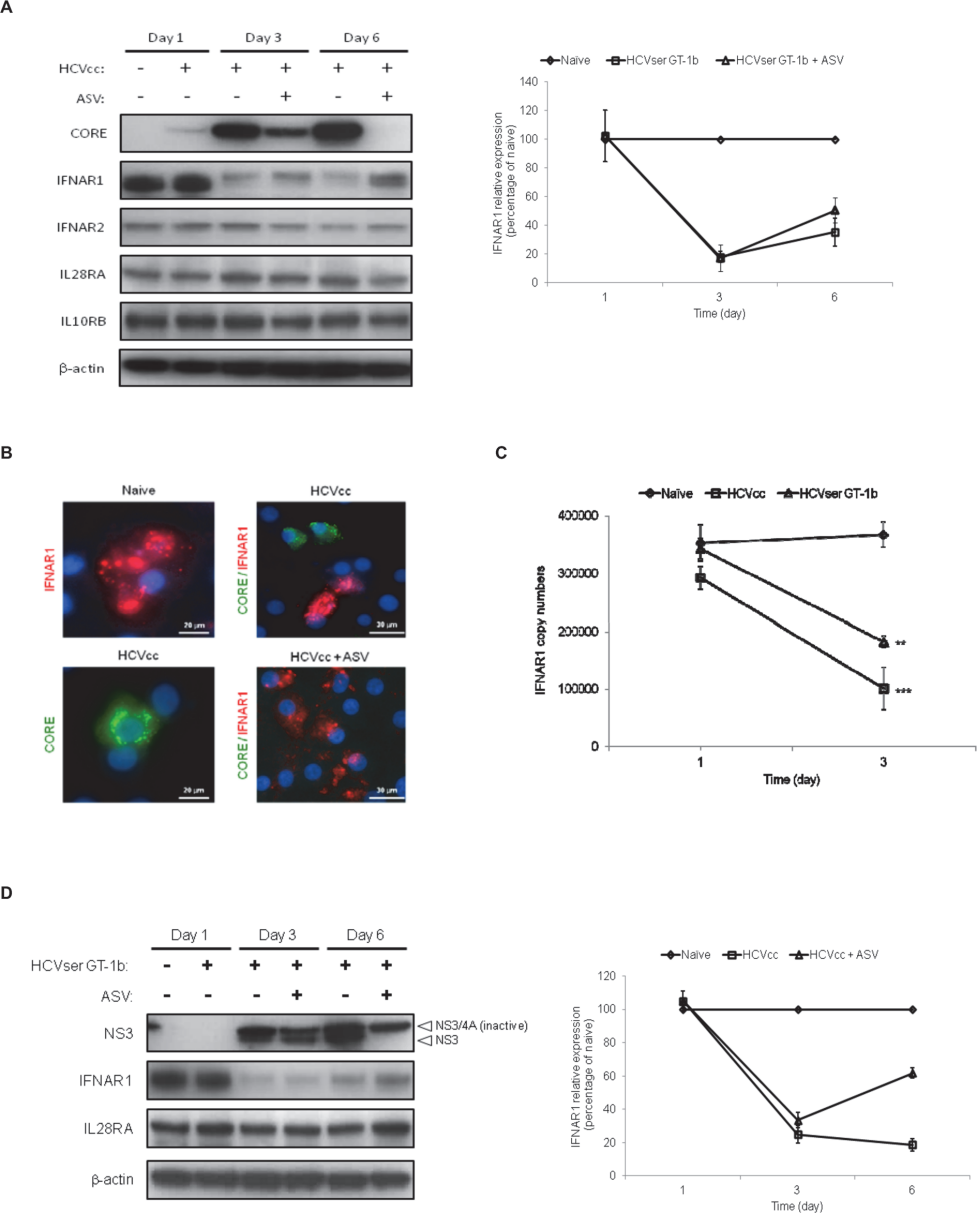
Productive HCV replication represses IFNAR1 expression in infected hepatocytes. iHLCs were infected with HCVcc (MOI of 0.2), GT-1b HCVser or mock infected, and subsequently maintained in culture with medium replacement every 2 days. At 24 h post-infection, the NS3 PI ASV (0.5 μM) or vehicle control dimethyl sulfoxide (DMSO) was added to cell cultures during media replenishment. (A) Persistent HCVcc replication as measured by the detection of the virally-encoded core antigen and expression of type I (IFNAR1, IFNAR2) and type III IFN (IL28RA, IL10RB) co-receptor subunits in cells were monitored by Western immunoblotting at the indicated time points, with β-actin used as loading control. Right panel; Relative intensity of IFNAR1 detected in HCVcc infected cells maintained in the presence (open triangle) or absence (open square) of ASV treatment was quantified by densitometry analysis, normalized to β-actin levels in each sample, and expressed as a percentage relative to mock-infected cells. Data are representative of three independent Western immunoblot analyses. (B) Co-localization of IFNAR1 and HCV-core positive iHLCs were assessed by fluorescence microscopy. On Day 6 post-infection, naive and HCVcc-infected iHLCs were fixed, permeabilized and stained with antibodies to IFNAR1 (red) and HCV core antigen (green) as indicated. Nuclei were counterstained with Hoechst dye (blue) as shown in the overlays. (C) iHLC cultures infected in parallel with GT-1b HCVser or HCVcc were harvested at the indicated time points and IFNAR1 copy numbers estimated by quantitative RT-PCR following normalization to cellular GAPDH levels in each sample. Results are expressed as mean ± standard deviations (n = 4). Statistical analysis was performed by Bonferroni’s multiple comparison tests: **, *P* < 0.05; ***, *P* < 0.001. (D) Cell lysates were harvested at the indicated time points and protein levels of IFNAR1 and IL28RA were examined by Western immunoblotting. Susceptibility of HCVser to the NS3 PI ASV was determined using an antibody directed against NS3. Arrows indicate the presence of the processed and unprocessed forms of the HCV-encoded NS3/4A protease in infected iHLCs. Detection of β-actin served as loading control. Right panel; Relative intensity of IFNAR1 detected in HCVser infected cells maintained in the presence (open triangle) or absence (open square) of ASV treatment was quantified by densitometry analysis, normalized to β-actin levels in each sample, and expressed as a percentage relative to mock-infected cells. Data are representative of two independent Western immunoblot analyses.

To understand the significance of down-regulation of IFNAR1 transcript by HCVcc, protein expression of type I and type III co-receptor subunits in infected hepatocytes was monitored. Since PHH are difficult to maintain in culture and support only low levels of HCV replication, human-induced hepatocyte-like cells (iHLCs) derived from pluripotent stem cell technology were used in these analyses. These cells offer a physiologically relevant *in vitro* model to study innate responses with the capacity to support persistent HCV replication.[38, 39] Using quantitative RT-PCR, HCVcc RNA levels in infected iHLCs were shown to gradually increase over 9 days in culture, whereas low levels were noted in PHH after 3 days of infection (S1 Fig.). HCV core protein was detected in iHLCs by Western immunoblotting 24 h after viral infection (Fig. 2A), and continuous replication was confirmed over long-term maintenance in culture. Up-regulation of ISGs such as OAS1, MX1, and the transcriptional regulator ISGF3γ were observed within 24 h of infection in iHLCs (S1 Fig., panel D; S2 Table). In contrast, UV-irradiated HCVcc failed to stimulate gene expression as previously reported.[19] Reduced expression of the IFNAR1 protein (19% relative to mock-infected cells; Fig. 2A, right panel) was readily observed by Day 3 post-infection in HCVcc-infected iHLCs and appeared dependent on productive viral replication. Indeed, cell cultures treated with the NS3 PI ASV after viral infection exhibited decreased HCV core antigen production which correlated with the restoration of IFNAR1 synthesis by Day 6 (62% expression relative to mock-infected cells; Fig. 2A, right panel). Conversely, the type III IFN co-receptor subunit IL28RA was not affected in this model, highlighting the potential for differential regulation of IFN receptor expression by HCV infection. Consistent with these results, immunostaining analysis demonstrated the lack of co-localization between IFNAR1 protein and HCV-core positive cells, whereas ASV-treated cultures displayed homogeneous IFNAR1 staining (Fig. 2B).

To further validate the HCV-induced repression of IFNAR1, iHLCs were infected in parallel with high titer HCV GT-1b derived from patient serum (HCVser) or with HCVcc. In agreement with our microarray analysis in PHH, down-regulation of the IFNAR1 transcript was observed in HCVcc-infected iHLCs by Day 1 post-infection, becoming significantly more apparent (*P* < 0.001) by Day 3 (Fig. 2C). Similarly, IFNAR1 transcription was significantly decreased by Day 3 post-infection with GT-1b HCVser (*P* < 0.05), and was further associated with reduced protein expression in cells. This phenotype was concomitant with HCVser replication as documented by Western immunoblotting analysis. Infection with GT-1b HCVser was less robust than with HCVcc (data not shown), but clearly sensitive to ASV inhibition which selectively targets the active site of the NS3 protease leading to inhibition of polyprotein processing. As a result, the processed form of HCV NS3 protease still detectable in infected iHLCs 2 days following ASV treatment, was not visible on Day 6 post-infection (Fig. 2D). The presence of NS3/4A complex on Day 6, albeit at a lower level compared to ASV-untreated HCVser infected cells, is a consequence of its long intracellular half-life.[40] Inhibition of HCVser replication correlated with an increased expression of IFNAR1 protein by Day 6 (51% expression relative to mock-infected cells; Fig. 2D, right panel). Furthermore, as was documented with HCVcc, the level of IL28RA receptor subunit was not reduced by HCVser replication (Fig. 2D). Overall, these results strongly suggest that HCV infection selectively impairs expression of IFNAR1 in hepatocytes.

### Stimulation of the Jak-STAT pathway by alfa, but not Lambda, is altered in HCV-infected iHLCs

**Fig 3:**
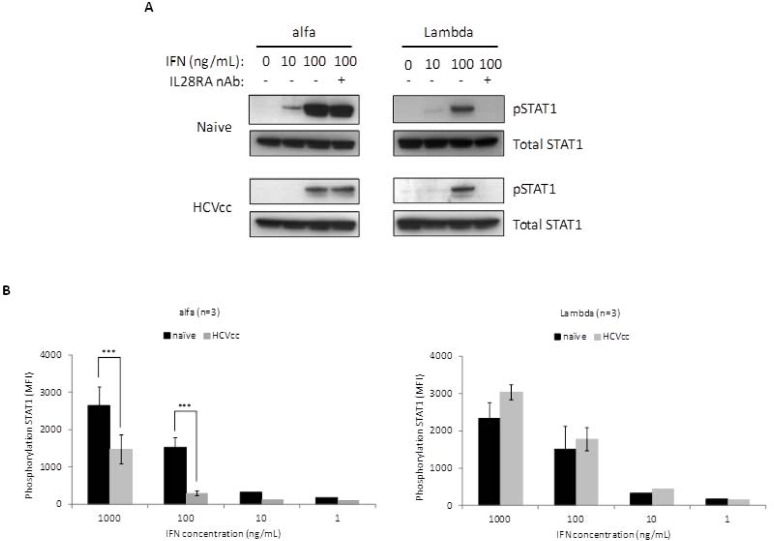
Impairment of alfa signaling in HCVcc-infected hepatocytes. (A) Naive and HCVcc infected iHLC cultures maintained in presence or absence of the IL28RA nAb were treated for 15 minutes with 10 ng/mL or 100 ng/mL of alfa or Lambda. Cell lysates were then prepared, and equal amounts of proteins subjected to Western immunoblotting to examine the levels of STAT1 phosphorylation using an antibody directed against phospho STAT1 (pSTAT1; Tyr701). Detection of total STAT1 served as loading control to ensure that equivalent amounts of protein were analyzed among samples. (B) Phosphorylation of STAT1 in iHLCs was evaluated upon stimulation using the Luminex bead-based assay. MFI values were reported as mean values of three independent cultures. Error bars show the standard deviations. Two-way ANOVA statistical analysis was performed using Bonferroni post test: ***, *P* < 0.001.

Although the affinity of IFN-α to IFNAR1 is weak, its interaction is critical for the efficient assembly and functional stability of the ternary ligand-receptor complex.[41, 42] Hence, Jak-STAT signaling induced by alfa or Lambda was examined in iHLCs following HCVcc infection. Signal transduction through engagement of type I or type III IFNs to their distinct receptor complexes leads in both cases to activation of *Janus* kinases. This triggers the phosphorylation of STAT1 and STAT2 molecules, essential for their subsequent nuclear translocation as part of active homo- and hetero-dimeric complexes. Consistent with previous findings, alfa induced greater phosphorylation of STAT1 in naive iHLCs compared to Lambda at similar concentrations (Fig. 3A). However, the induction of STAT1 phosphorylation by alfa (10 or 100 ng/mL) was attenuated in HCVcc-infected iHLCs, whereas Lambda signaling remained efficient. Since our transcriptional analysis indicated that HCVcc infection induced expression of type III IFNs in iHLCs, it was necessary to rule out the effects of endogenous IFN-λ on the activation of STAT1. Therefore, cell cultures were treated for 1 h prior to HCVcc infection and further maintained in the presence of a neutralizing antibody (nAb) directed against the extracellular domain of the IL28RA receptor subunit (IL28RA nAb). This antibody inhibits activation of the Jak-STAT pathway by all 3 recombinant (r) forms of IFN-λ, but has no effect on the biological activity of rIFN-α (S3 Fig.). The IL28RA nAb completely abrogated Lambda stimulation (100 ng/mL) of STAT1 phosphorylation in naive cells and HCVcc-infected iHLCs. Conversely, pre-incubation of cells with the IL28RA nAb had no significant effect on STAT1 phosphorylation upon stimulation with alfa (100 ng/mL) (Fig. 3). This suggests that the level of endogenous IFN-λ produced in the HCVcc-infected iHLC culture is not the cause of the reduction in alfa signaling upon infection. The effect of HCVcc infection upon IFN-stimulated STAT1 phosphorylation was also quantitated using the Luminex bead array technology (Fig. 3B). Relative to naive iHLCs, reduction of approximately 50% and 80% in measurable phosphorylated STAT1 were significant (*P* < 0.001) during exposure to high concentrations of alfa (1000 and 100 ng/mL, respectively), whereas no effect of HCVcc infection was observed on Lambda signaling.

### Lambda induction of ISG expression in HCV-infected iHLCs

**Fig 4:**
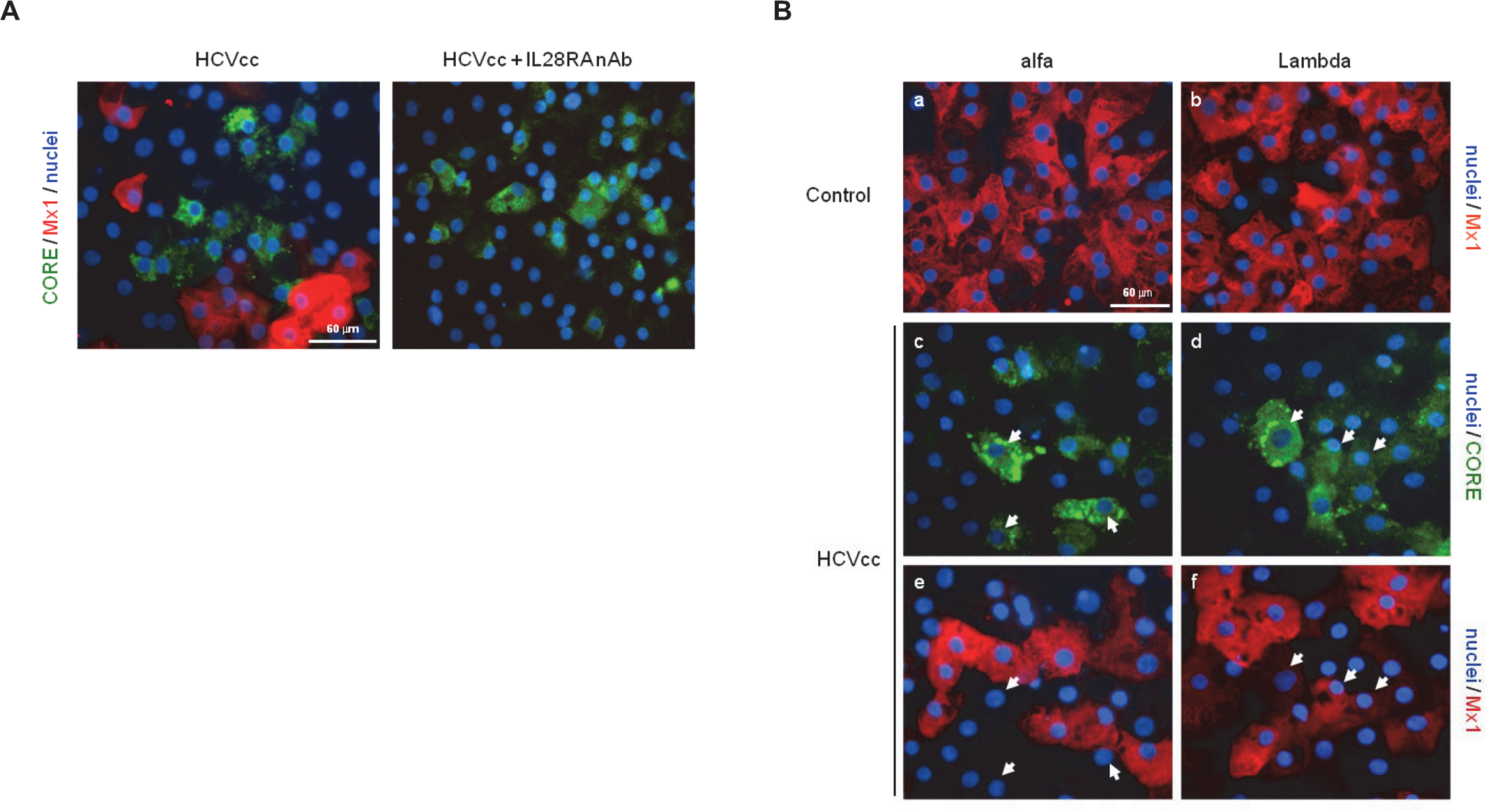
Lack of response to alfa treatment in HCV-infected hepatocytes. (A) Expression of Mx1 protein (red) and HCV-core antigen (green) was monitored in HCVcc-infected iHLC cultures maintained in presence or absence of the IL28RA nAb (10 μg/mL). Dual immunostaining was performed as described in the legend for Fig. 2. Nuclei were counterstained with Hoechst dye (blue) as shown in the overlays. (B) Induction of Mx1 expression (red) was assessed in Naive iHLCs 24 h following stimulation with 10 ng/mL of alfa (a) or Lambda (b). iHLC cultures infected with HCVcc were maintained in the presence (left panels) or absence (right panels) of the IL28RA nAb (10 μg/mL). On Day 6 post-infection, cells were treated as indicated with 10 ng/mL of alfa or Lambda for 24 h, and then dual immunostaining was performed using antibodies directed against Mx1 (red) and the HCV-core antigen (green). Arrows indicate examples of different HCV-Mx1 co-localization patterns in overlaid optical field. Scale bar, 60 μm.

The effect of HCV on the hepatic innate responses was next investigated using the immunocompetent iHLC model. Induction of the ISG protein myxovirus resistance protein 1 (Mx1, also known as MxA), a key biomarker of the innate response against HCV, was assessed in iHLCs following infection with HCVcc. Immunostaining analysis revealed the detection of HCV-core positive cells predominantly in clusters within the cultures (Fig. 4A). This observation is in agreement with recent *in situ* hybridization RNA analysis demonstrating clustered spatial distribution of HCV-infected cells in liver biopsies of chronically infected patients, signifying cell-to-cell spread as the predominant mode of viral transmission.[43] Interestingly, expression of Mx1 was mainly detectable in uninfected iHLCs surrounding HCV-positive clusters. In contrast, Mx1 protein was not visible in HCVcc-infected iHLCs cultures treated with the IL28RA nAb, strongly implicating endogenous IFN-λs produced during infection as the main stimulus driving ISG induction in this hepatic system. Production of IFN-λ-1 (average peak of 25 pg/mL) by HCVcc-infected iHLCs was confirmed in our experiments (S4 Fig.), with no release of IFN-α or IFN-β (data not shown).

It was previously shown that ISG proteins are poorly or not induced in HCV-infected hepatoma Huh-7 cells after rIFN-β treatment, despite efficient stimulation at the transcriptional level.[44] As seen in Fig. 4B (panels a-b), alfa and Lambda treatments induced the expression of Mx1 protein to similar degrees in naive iHLCs. However, in HCVcc-infected cell cultures maintained in the presence of the IL28RA nAb, alfa treatment did not induce Mx1 expression in HCV-core positive iHLCs, whereas surrounding uninfected cells were responsive to stimulation (Fig. 4B, panels c-d). In contrast, Lambda treatment induced the expression of Mx1 in HCVcc-infected iHLCs reflecting the onset of the innate response (Fig. 4B, panels e-f). In line with these observations, the absence of active phosphorylated STAT1 was noted in the nucleus of HCVcc-infected iHLCs treated with alfa, but not with Lambda (S5 Fig.). Overall, these findings imply that type III IFN production in response to HCV infection provides an antiviral state limiting the spread of infection, and that infected hepatocytes remain susceptible to exogenous Lambda, but not to alfa treatment.

### Lambda prolongs induction of distinct biological pathways in hepatocytes

**Fig 5:**
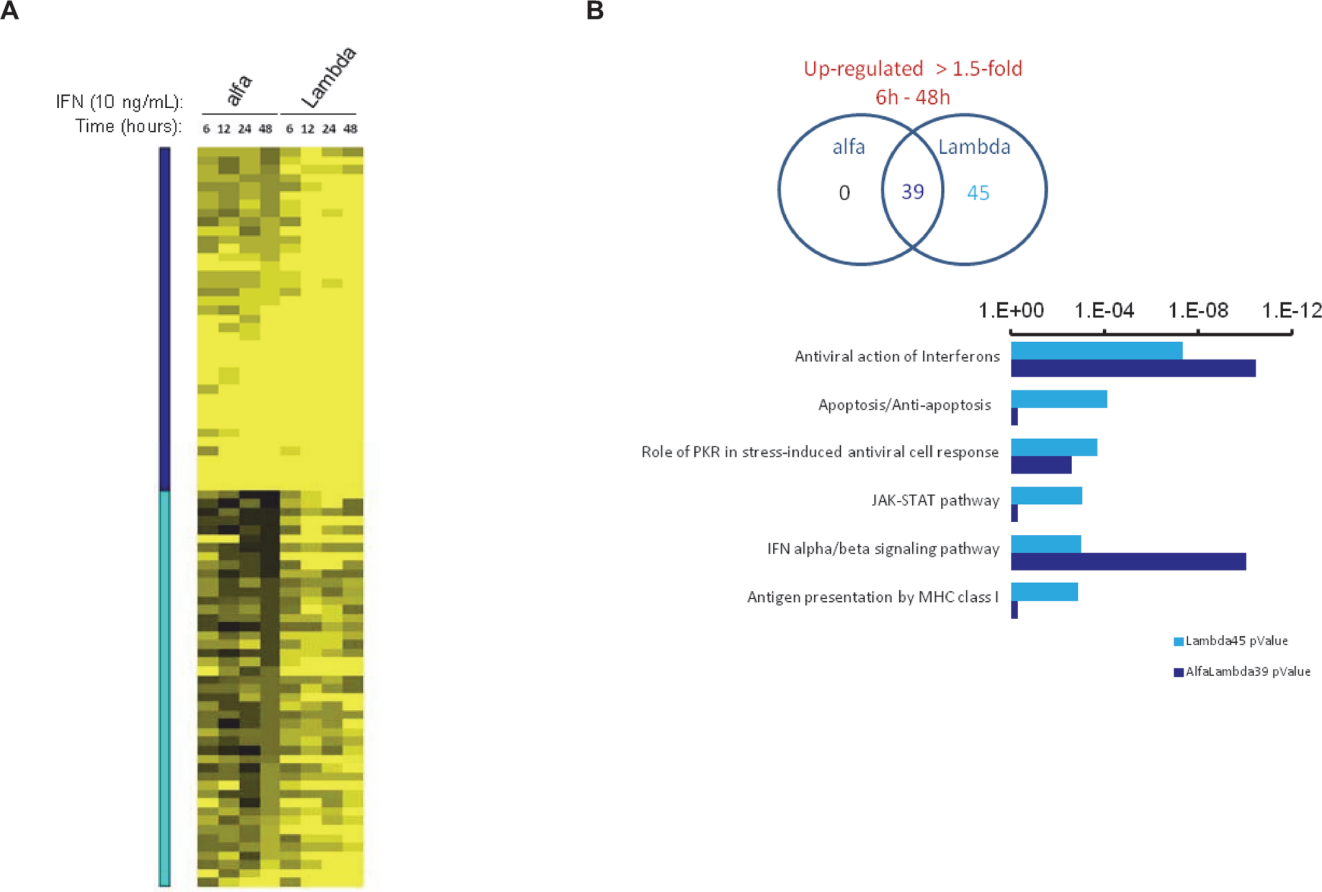
Lambda induces broader and more sustained biological activities than alfa in naive hepatocytes. Microarray transcriptional profiling of naive iHLCs treated with 10 ng/mL of alfa or Lambda over the course of 48 hours. (A) Relative expression of 83 genes shown to be up-regulated >1.5-fold at all time points by one or both agents, normalized to untreated iHLC at the same time point. Genes indicated by dark blue overlay are consistently regulated by both agents throughout the time series, while genes indicated by light blue overlay show more sustained induction with Lambda compared to alfa. (B) Venn diagram displaying numbers of genes up-regulated (>1.5-fold change) following the indicated treatments. Significance values from MetaCore network analysis for the top six signaling pathways related to the 45 genes with sustained up-regulation by Lambda, and their corresponding *P* value for the list of 38 shared genes.

Type III IFNs have been shown to induce a more sustained induction of Jak-STAT signaling than IFN-α, albeit with a relatively lower magnitude of ISG expression.[45] To further characterize hepatic ISG induction upon exposure to the two IFNs, we performed transcriptional profiling of naive iHLCs treated with 10 ng/mL of alfa or Lambda for 6, 12, 24 or 48 hours. By filtering for transcripts that were up-regulated by greater than 1.5-fold at all time points by one or both agents, 83 distinct genes were identified. Lambda consistently up-regulated all 83 genes by ≥ 1.5-fold throughout the treatment time-course whereas only 38 of these genes were up-regulated after treatment with alfa (Fig. 5A, S3 Table). For the shared 38 genes, the magnitude of induction by Lambda was stronger at all time points. The 83 regulated genes were examined for their representation of defined biological processes using MetaCore network analysis (Fig. 5B). While both alfa and Lambda consistently up-regulated transcripts for proteins with roles related to antiviral activity, the top six signaling pathways relating to the 45 genes with stronger and more sustained induction by Lambda indicated additional regulated biological processes including apoptosis, Jak-STAT signaling and antigen presentation.

## Discussion

Our data provide important novel insights into the mechanisms of responsiveness to IFN-based therapies. It has previously been shown that the level of short-lived IFNAR1 protein can create a rate-limiting step in the formation of the ternary ligand-receptor complex, and that depletion or functional disruption of IFNAR1 in cells leads to a significant reduction in type I IFN signaling.[46, 47] In this study, we demonstrated that down-regulation of IFNAR1 following HCV infection had a significant impact on the capacity of infected primary hepatocytes to respond to alfa. Additionally, treatment of HCV-infected cultures with the NS3 PI ASV restored expression of IFNAR1 in iHLCs, indicating that viral replication was required for the altered phenotype. Conversely, transient up-regulation of the unique type III IFN co-receptor IL28RA occurred early after HCV infection, and infected iHLCs remained susceptible to Lambda treatment. In agreement with these findings, Chandra *et al*. recently showed in an HCVcc-infected hepatoma cell model that viral replication induces endoplasmic reticulum stress and that an autophagy response is responsible for the degradation of IFNAR1 ultimately affecting IFNα but not IFN-λ signaling.[48] A number of studies have reported lower levels of type I receptor transcriptional expression in liver biopsies from patients with chronic HCV infection who exhibit non-responsiveness to alfa-based therapy.[49–51] Recently, endogenous levels of Type III IFNs were suggested as being responsible for the partial restoration of IFNAR1 transcriptional expression in peripheral blood mononuclear cells from HCV patients with favorable *IL28B* genotype.[52] In our work, successful infection in the iHLC culture system using clinical viral isolates further substantiated the physiological relevance of the inverse modulation of IFNAR1 mediated by HCV in patients. However, conclusive validation of IFN receptor modulation by HCV in clinical settings remains challenging given the chronic phase of the disease and the difficulties in reliably interpreting hepatic protein expression profiles in patients relative to those defined in healthy subjects.

Virologic failure to an alfa plus RBV regimen in HCV infected patients is not attributed to the emergence of drug resistant variants. Instead, a combination of several factors, including host genetic variations and viral determinants, has been shown to influence treatment outcome in patient populations representing GT-1 through 4. Notably, several studies have indicated that patients with high baseline viral load (>800,000 IU/mL) are less likely to achieve SVR following alfa-based therapy.[25, 26] Early dynamics of viremia following alfa treatment are also predictive indicators of subsequent outcome. A robust biphasic HCV RNA decline is essential for optimal outcome to alfa-based therapies. This has been used to explain the increased response rates observed in GT-2 patients compared to GT-1.[27] In our study, down-regulation of IFNAR1 was observed in iHLCs following infection with HCV derived from GT-3a patient serum (S2 Fig.). This is interesting given the increasing evidence that HCV GT-3 may not be such an easy-to-treat patient population as previously assumed.[53] Retrospective analyses of alfa-based therapy trials indicate that SVR rates for GT-3 patients are closer to those infected with GT-1. Similarly, reduced efficacy against GT-3 has been observed with new pan-genotypic DAAs during clinical development.[54] In Phase 2b trials, SVR_24_ rates for GT-3 were comparable to GT-2 among patients receiving Lambda plus RBV (GT-3: 57–83%, GT-2: 58%-71%), but were lower in the alfa arm (GT-3: 40%, GT-2: 67%).[24] Therefore beyond our study, infection of iHLCs with clinical isolates representing various genotypes may have broader significance in understanding non-responsiveness to alfa- and DAA-based therapies.

Immunostaining analysis addressed the antiviral effectiveness of alfa and Lambda in HCV-infected hepatocytes. Since HCV induces expression of endogenous type III IFNs, the use of an IL28RA nAb that abrogates type III IFN signaling was essential to selectively block autocrine and paracrine stimulatory effects. This approach revealed the failure of alfa to induce Mx1 protein in HCV-infected iHLCs, in part due to the down-regulation of IFNAR1 and the inability to stabilize functional receptor complexes. In agreement, Garaigorta *et al*. previously reported that protein expression of ISGs (Mx1 and USP18) are poorly induced in HCV-infected Huh-7 cells following stimulation with IFN-β which also utilizes the type I IFN receptor complex.[44] Conversely, Lambda stimulated Mx1 expression in those cell populations further suggesting that HCV does not interfere directly with ISG transcription or translation during viral replication. The prolonged capacity of Lambda to stimulate hepatic innate responses, as documented herein, may have contributed to the more rapid declines in HCV RNA observed in patients treated with Lambda compared to alfa. Early *in vitro* profiling analyses have indicated that the kinetics and magnitude of signal transduction in hepatoma cell lines differ following stimulation with type I and type III IFNs, albeit that both induce an overlapping repertoire of ISGs.[55, 56] Indeed, the overall ISG induction pattern in naive PHH upon alfa treatment tends to peak early (within 8 hours) followed by a rapid decline, whereas Lambda triggers a lower magnitude but prolonged induction in ISG expression.[57] Our transcriptional analysis of ISG induction within naive iHLCs identified a more sustained pattern for Lambda, resulting in the activation of a distinct set of genes as compared to those induced by alfa. Several of these transcripts are known ISGs encoding proteins involved in apoptosis (e.g. TNFSF10), signaling transduction (e.g. STAT1), and antigen presentation by major histocompatibility complex class I molecules (e.g. TAP1). The repertoire of genes with sustained up-regulation by Lambda may therefore promote biological processes which are additionally beneficial to the elimination of infected hepatocytes. Moreover, treatment with rIFN-λ-1 has been shown to enhance the function of immune cells such as myeloid and plasmacytoid dentritic cells by increasing the amount of IFN-α and IFN-λ production in either an autocrine or paracrine manner.[58, 59] Although it warrants further investigation, these findings suggest that Lambda may induce changes in host immune functions which are important for bridging the intrahepatic innate response to the onset of adaptive immunity crucial in the resolution of HCV infection.

It is well-established that patients with a pre-activated endogenous IFN system at baseline respond poorly to an alfa plus RBV regimen.[60] However, the cellular source and type of IFN driving continuous ISG expression in patients chronically infected with HCV have not been defined. In a recent study, Wieland *et al*. demonstrated by *in situ* hybridization that clusters of infected cells and neighboring cells in liver biopsies from patients have increased levels of ISG transcripts strongly suggesting that the stimulus driving their expression originates from infected hepatocytes. By using the immunocompetent iHLC model, we extended these observations by showing that endogenous production of type III IFNs upon HCV infection resulted in the stimulation of Mx1 protein expression in uninfected cells surrounding clusters of infected cells. The lack of Mx1 detection in HCV-infected cultures maintained in the presence of the IL28RA nAb further confirmed the role of type III IFNs as main drivers of ISG expression in iHLCs. Collectively, these findings have important ramifications in defining the dynamics of the intrahepatic innate antiviral response. Following HCV infection, hepatocytes would be the most likely source of type III IFN production, thereby creating an antiviral environment for neighboring uninfected cells, and limiting the spread of infection within the liver. This hypothesis is supported by the clustered spatial distribution of HCV-infected cells noted herein, and previously described by Wieland *et al*. in liver biopsies obtained from chronically HCV-infected patients.[43] Furthermore, the level of IFN-λ-1 released in HCV-infected iHLC cultures is sufficient to inhibit de novo infection in naive hepatocytes.[19] In patients, endogenous IFN-λs may therefore induce an environment within uninfected hepatocytes that is refractory to further stimulation by alfa, which may thus impact treatment outcome.

Negative feedback loops differentially govern type I and type III signaling, relevant to the establishment of antiviral immunity. The IFN-mediated activation of the Jak-STAT pathway is tightly regulated by various cellular factors such as SOCS family members, USP18, the protein phosphatase 2A (PP2A), and protein inhibitors of STAT (PIAS).[60] Along those lines, SOCS1 and SOCS3 transcriptional expression in hepatocytes remains detectable for only a short period of time upon alfa treatment indicating that by inhibiting *Janus* kinases both proteins are likely responsible for early termination of STAT activation.[17] In addition, the sustained up-regulation of USP18 was associated with a long-lasting refractory state to IFN-α stimulation in hepatocytes and other primary human cells.[18] The specific interaction of USP18 with IFNAR2 selectively affects IFN—induced signaling while having a marginal effect on other classes of IFNs, including type III IFNs. Therefore, it is interesting to note that both SOCS1 and USP18 transcripts were up-regulated in hepatocytes during HCV infection (S1 Table). Their up-regulation is most likely associated with endogenous IFN-λ signaling, given the lack of IFN-α production in our HCV-infected cell cultures. Further work will be required to understand the duration of type III IFN release during HCV infection as well as the scope of their biological activities, since the HCV-encoded NS3/4A protease may ultimately block RIG-I and TLR3 pathways to modulate type III production from infected hepatocytes.[61, 62]

In conclusion, we provide additional information on the ability of HCV to impede stimulation of JAK-STAT signaling by alfa but not by Lambda in infected primary hepatocytes. Our findings further highlight the modulation in expression of type I and type III IFN receptors in the liver during the course of infection, potentially crucial in the outcome of IFN-based therapy. Early robust virological responses observed in patients upon Lambda treatment may be a reflection of its enhanced capacity to induce innate responses in infected and uninfected hepatocytes that are otherwise non-responsive to alfa treatment. Finally, our data support for the first time a model depicting the production of endogenous type III IFNs from HCV-infected hepatocytes as the main stimulus of ISG induction in the liver, which may be relevant in the refractory state of uninfected hepatocytes to alfa treatment and in the putative restoration of innate immunity by DAAs.

## Supporting Information

S1 Fig Characterization of HCVcc infection in human-induced hepatocyte-like cells (iHLCs). PHH or iHLCs were infected with HCVcc at an MOI of 0.2 as described in the Materials and Methods section, and subsequently maintained in culture with daily medium replacement. Cells were harvested at the indicated time points and total RNA isolated. Copies of HCV RNA were estimated in PHH (A) or iHLCs (B) by quantitative RT-PCR following normalization to cellular GAPDH levels in each sample. Data are representative of two independent experiments. (C) Viral replication of infectious and UV-irradiated HCVcc as measured by the detection of the virally-encoded core in cells was monitored by Western immunoblotting at the indicated time points with β-actin used as loading control. (D) Expression of OAS1, MX1 and ISGF3λ transcripts was monitored by RT-PCR in iHLC cells following inoculation with infectious or UV-irradiated HCVcc. Total RNA was isolated from cells at the indicated time points and amplification of cDNA was performed using gene-specific primers (System Biosciences). Mock-infected cells (naive) served as a control.
(TIF)

S2 Fig Infection of iHLCs with HCV derived from patient serum. Copies of Intracellular HCV RNA and IFNAR1 were estimated in iHLCs infected with a clinical viral isolate representing GT-3a. Cells were harvested at the indicated time points, total RNA isolated, and copy numbers were quantified by RT-PCR as described in the Materials and Methods section. Error bars represent standard deviation of three independent biological replicates.
(TIF)

S3 Fig Inhibition of type III IFN signaling by the IL28RA nAb. Human hepatoma Huh-7 cells that stably maintain the HCV subgenomic replicon representing GT-1a were treated for 1 h with the IL28RA nAb (10 μg/mL) as indicated, and cells were subsequently transfected with an interferon-stimulated response element (ISRE)-driven luciferase reporter plasmid (Agilent Technologies). The activation of the ISRE promoter in cells was determined in the presence or absence of (A) rIL-29, (B) rIL-28A, (C) rIL-28B (10 and 100 ng/mL) or (D) rIFN-α (10–1000 IU/ml) 24 h following stimulation. Relative light unit (RLU) values represent the average from three independent experiments. Error bars show the standard deviations.
(TIF)

S4 Fig Increased production of IL-29 in HCVcc-infected iHLC cultures. Supernatants from iHLC cultures infected with HCVcc (MOI = 0.2) were harvested at the indicated time points and levels of IL-29 were measured by enzyme-linked immunosorbent assay (ELISA) (eBioscience). The limit of detection of IL-29 in these experiments was determined to be 4.0 pg/mL (mean of three independent assays). Error bars show the standard deviations.
(TIF)

S5 Fig Lack of active phosphorylated STAT1 in HCVcc-infected iHLCs upon alfa treatment. (A) Expression of pSTAT1 was monitored in naive (control) and infected (HCVcc) iHLCs following stimulation with alfa or Lambda. iHLC cultures infected with HCVcc were maintained in the presence (left panels) or absence (right panels) of IL28RA nAb (10 μg/mL). On Day 6 post-infection, cells were treated with 10 ng/mL of alfa or Lambda for 1 h, and then immunostaining was performed as described in the legend for Fig. 2 using antibodies directed against pSTAT1 (red) and the HCV-core antigen (green). Nuclei were counterstained with Hoechst dye (blue) as shown in the overlays. Arrows indicate examples of different HCV-pSTAT1 co-localization patterns in overlaid optical field.
(TIF)

S1 Table Effect of HCVcc infection on gene expression in PHH. Representative probe sets for loci regulated in the innate immune response pathway: Fold-change relative to uninfected control cells. Data generated from 3 replicates.
(TIF)

S2 Table Effect of HCVcc infection on gene expression in iHLCs. Representative probe sets for loci regulated in the innate immune response pathway: Fold-change relative to uninfected control cells.
(TIF)

S3 Table Probe sets overview for the 83 loci whose expression was sustained in iHLCs during 48 hours upon stimulation with alfa or Lambda. (TIF)
