# Supplementary figures and images for: Impairment of Type I but Not Type III IFN Signaling by Hepatitis C Virus Infection Influences Antiviral Responses in Primary Human Hepatocytes
Source: PLoS One. 2015 Mar 31;10(3):e0121734. doi: 10.1371/journal.pone.0121734 (PMC4380495; doi:10.1371/journal.pone.0121734)

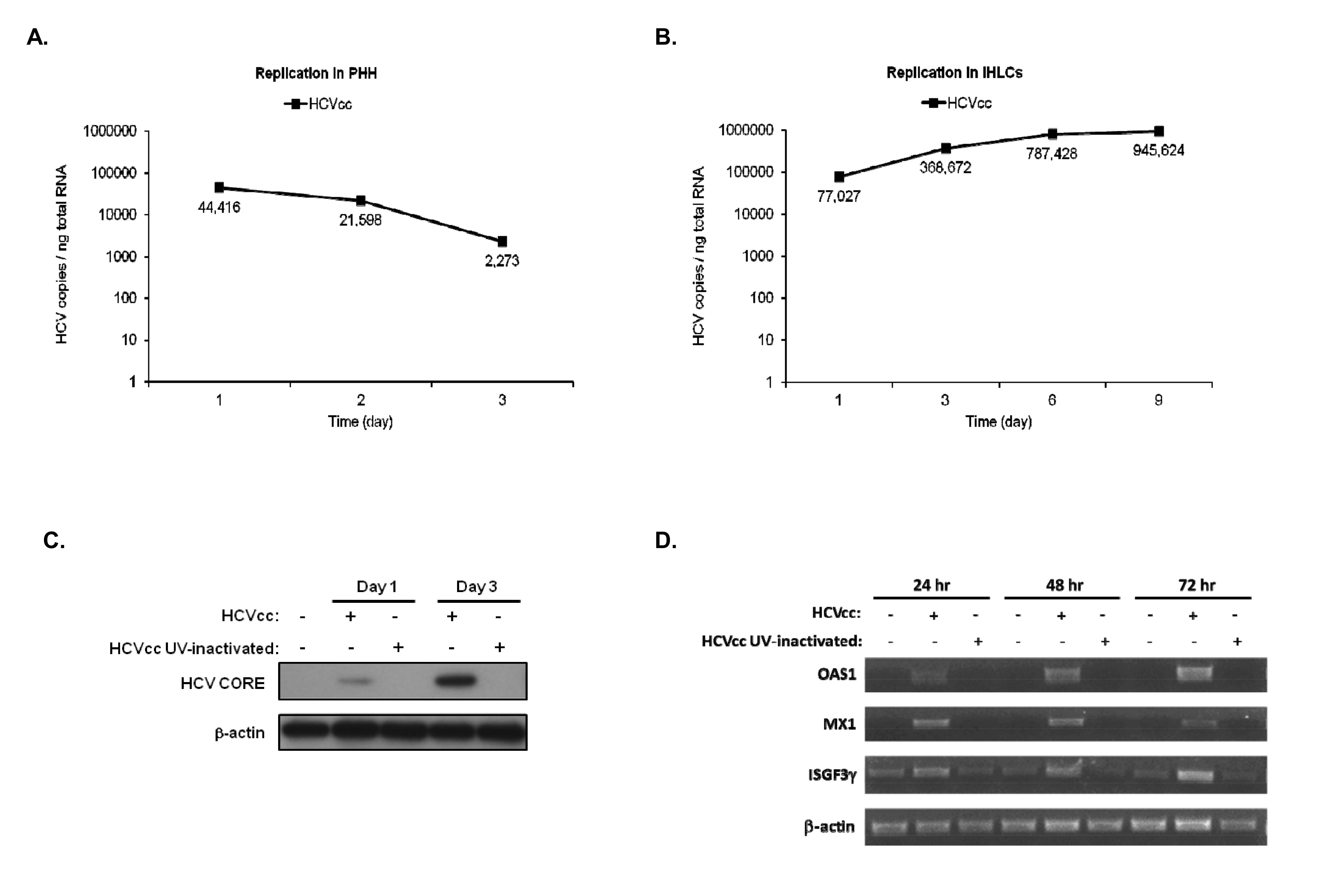

Supplement: S1 Fig — PHH or iHLCs were infected with HCVcc at an MOI of 0.2 as described in the Materials and Methods section, and subsequently maintained in culture with daily medium replacement. Cells were harvested at the indicated time points and total RNA isolated. Copies of HCV RNA were estimated in PHH (A) or iHLCs (B) by quantitative RT-PCR following normalization to cellular GAPDH levels in each sample. Data are representative of two independent experiments. (C) Viral replication of infectious and UV-irradiated HCVcc as measured by the detection of the virally-encoded core in cells was monitored by Western immunoblotting at the indicated time points with β-actin used as loading control. (D) Expression of OAS1, MX1 and ISGF3λ transcripts was monitored by RT-PCR in iHLC cells following inoculation with infectious or UV-irradiated HCVcc. Total RNA was isolated from cells at the indicated time points and amplification of cDNA was performed using gene-specific primers (System Biosciences). Mock-infected cells (naive) served as a control. (TIF) [file pone.0121734.s001.tif]

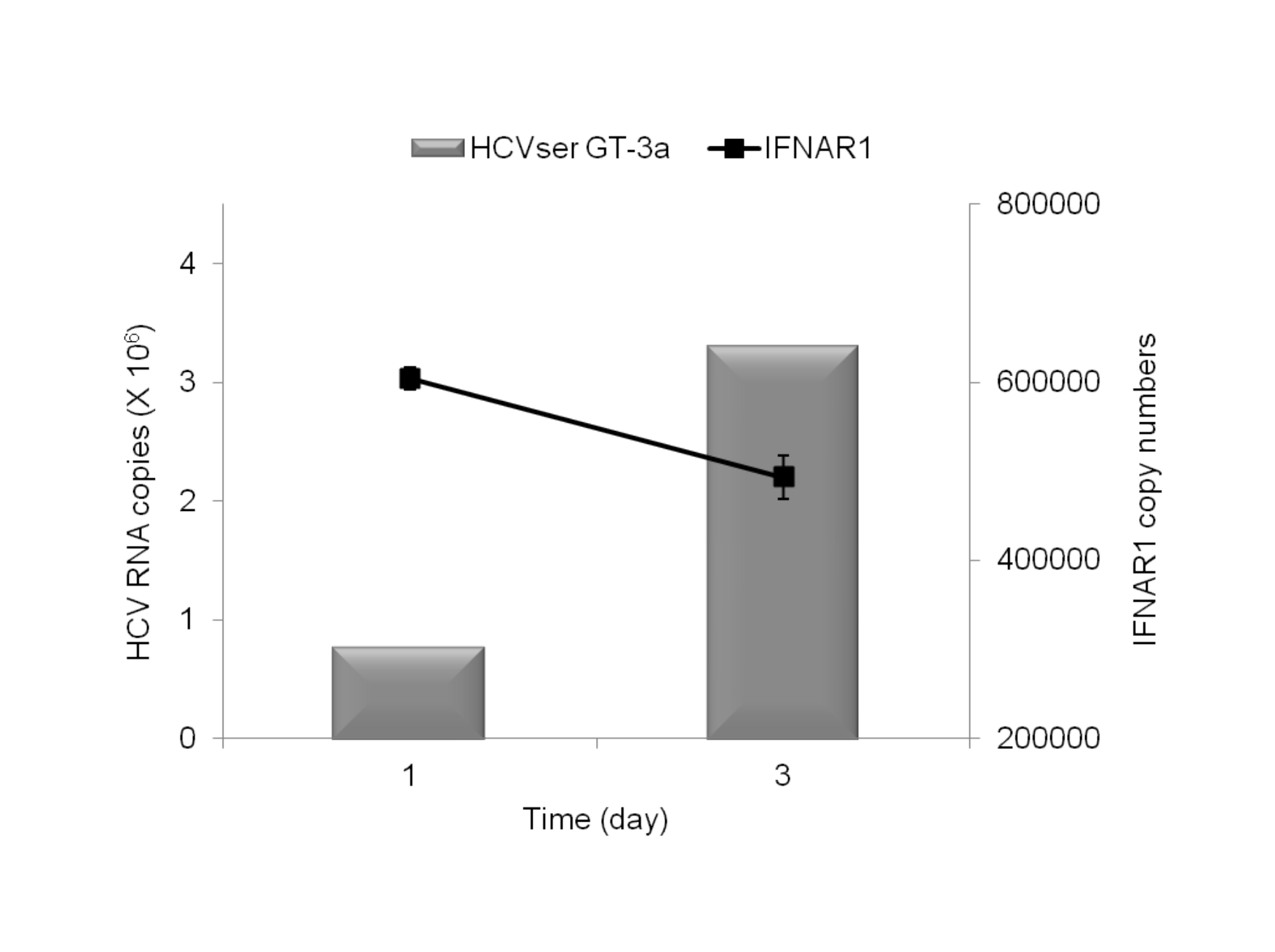

Supplement: S2 Fig — Copies of Intracellular HCV RNA and IFNAR1 were estimated in iHLCs infected with a clinical viral isolate representing GT-3a. Cells were harvested at the indicated time points, total RNA isolated, and copy numbers were quantified by RT-PCR as described in the Materials and Methods section. Error bars represent standard deviation of three independent biological replicates. (TIF) [file pone.0121734.s002.tif]

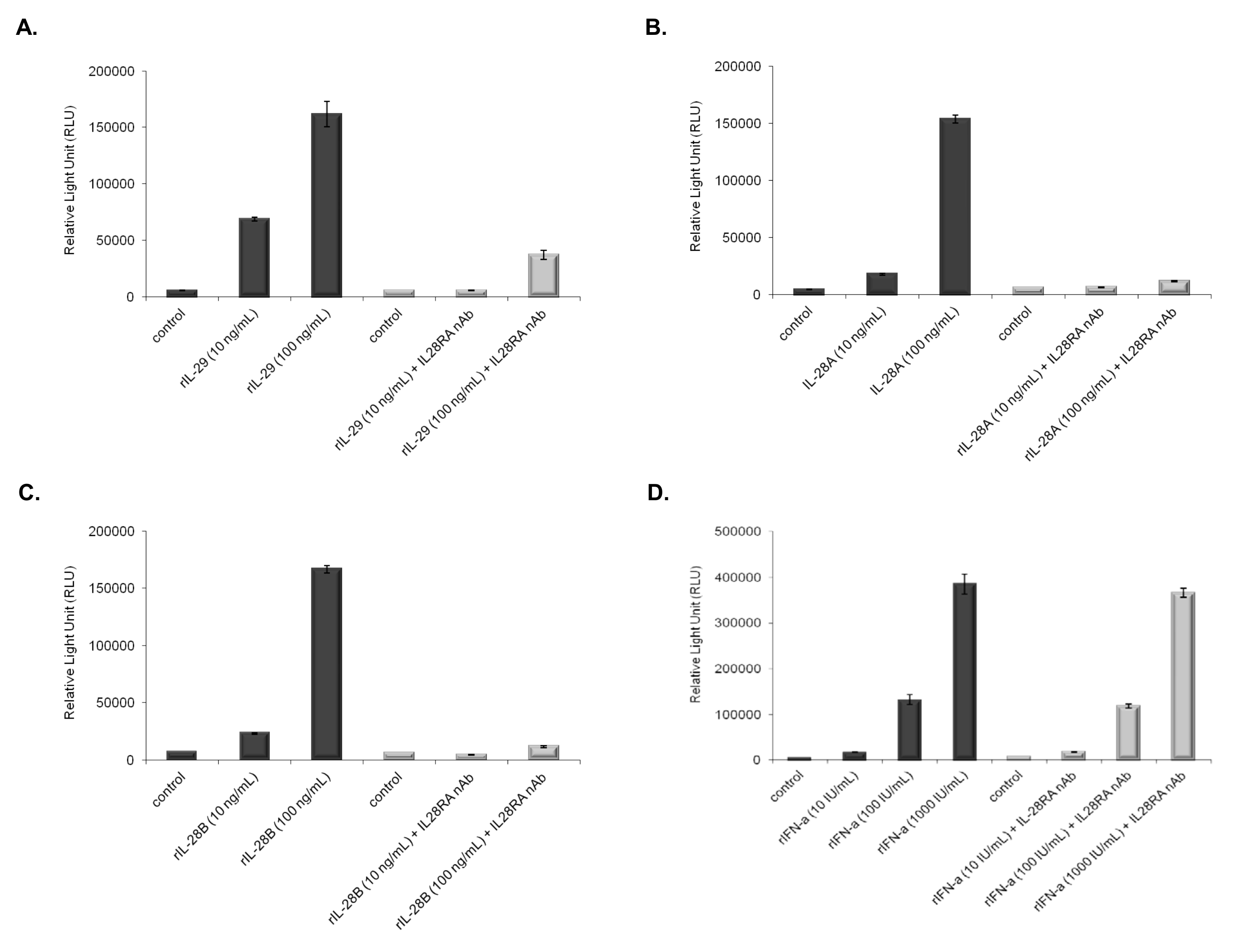

Supplement: S3 Fig — Human hepatoma Huh-7 cells that stably maintain the HCV subgenomic replicon representing GT-1a were treated for 1 h with the IL28RA nAb (10 μg/mL) as indicated, and cells were subsequently transfected with an interferon-stimulated response element (ISRE)-driven luciferase reporter plasmid (Agilent Technologies). The activation of the ISRE promoter in cells was determined in the presence or absence of (A) rIL-29, (B) rIL-28A, (C) rIL-28B (10 and 100 ng/mL) or (D) rIFN-α (10–1000 IU/ml) 24 h following stimulation. Relative light unit (RLU) values represent the average from three independent experiments. Error bars show the standard deviations. (TIF) [file pone.0121734.s003.tif]

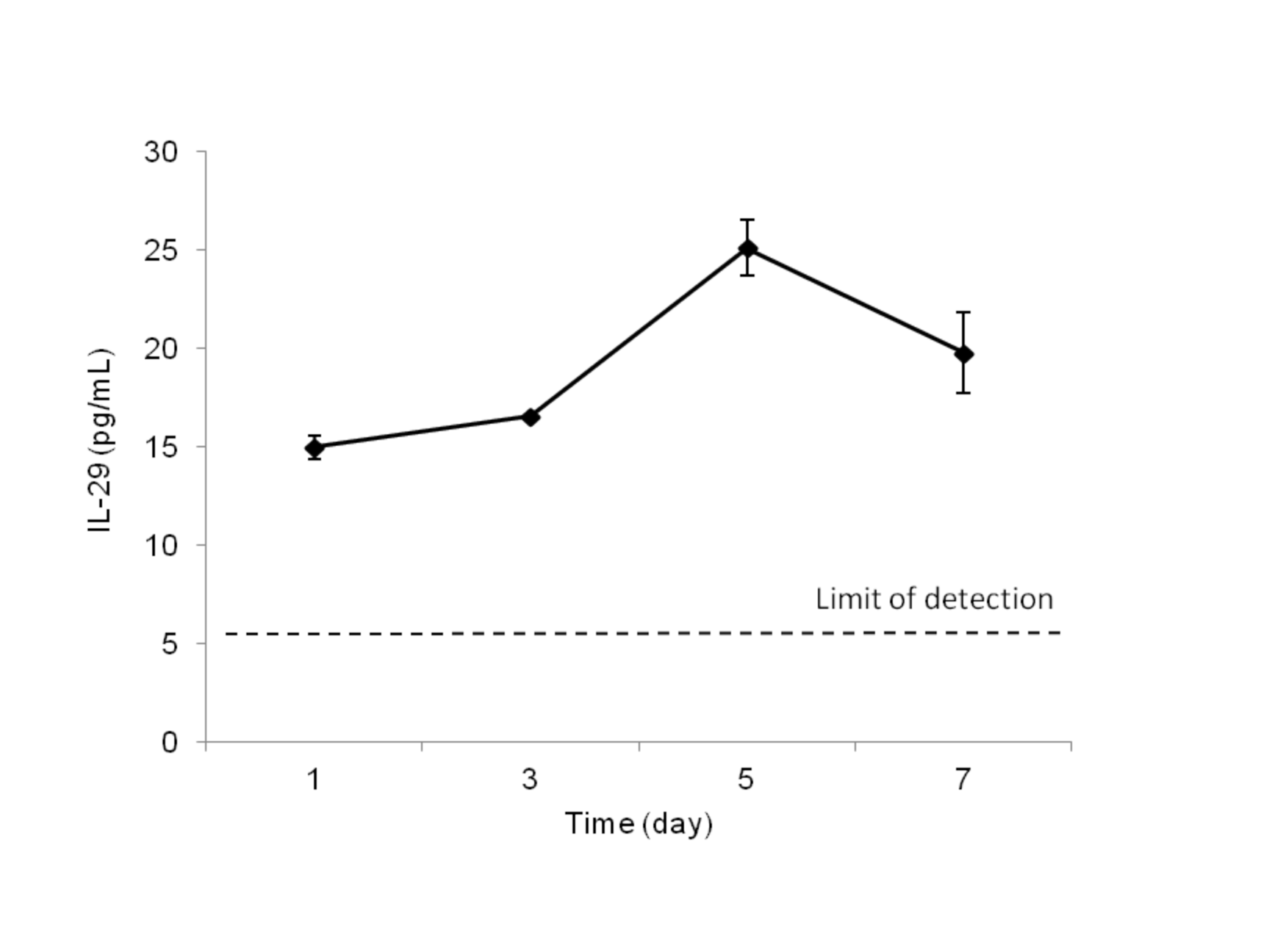

Supplement: S4 Fig — Supernatants from iHLC cultures infected with HCVcc (MOI = 0.2) were harvested at the indicated time points and levels of IL-29 were measured by enzyme-linked immunosorbent assay (ELISA) (eBioscience). The limit of detection of IL-29 in these experiments was determined to be 4.0 pg/mL (mean of three independent assays). Error bars show the standard deviations. (TIF) [file pone.0121734.s004.tif]

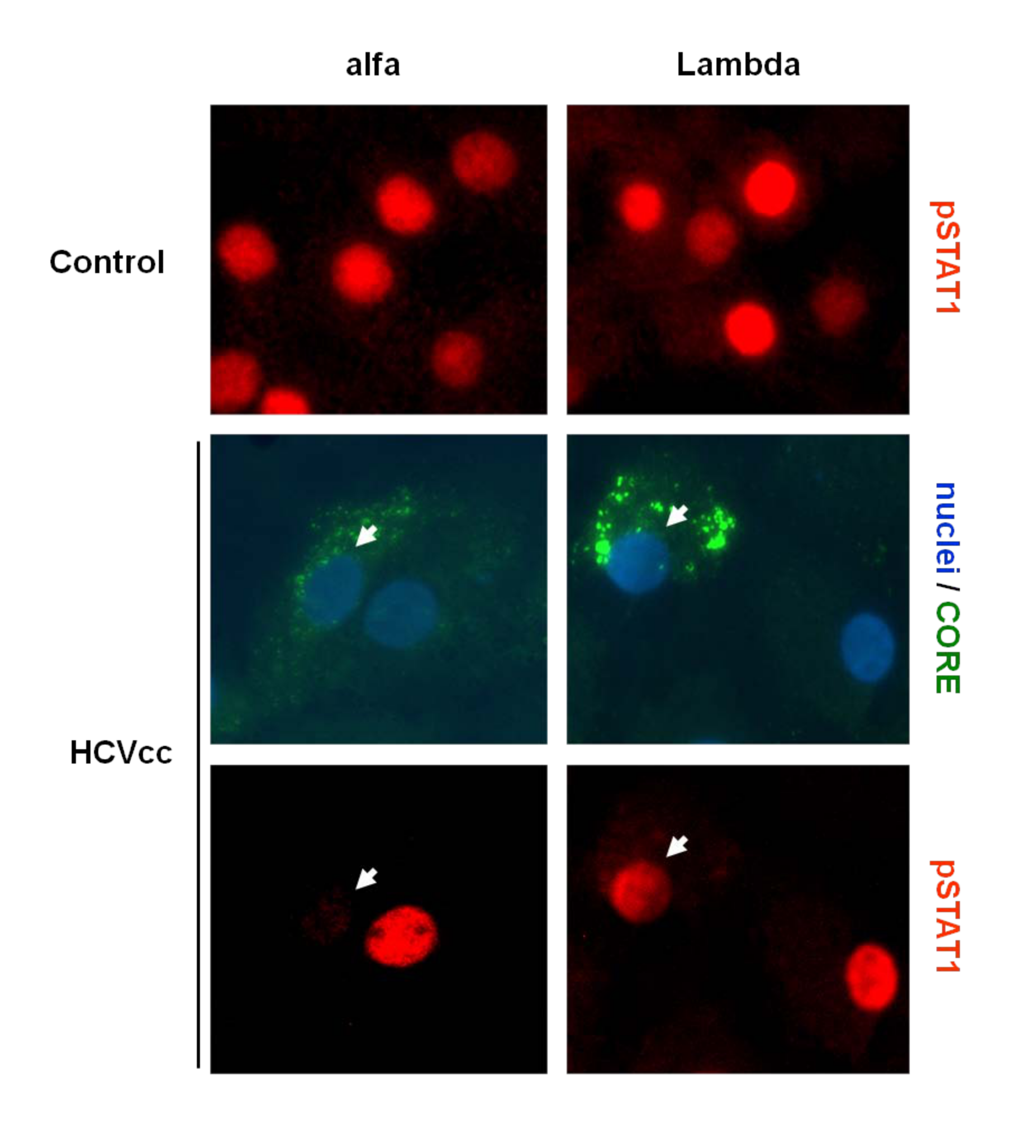

Supplement: S5 Fig — (A) Expression of pSTAT1 was monitored in naive (control) and infected (HCVcc) iHLCs following stimulation with alfa or Lambda. iHLC cultures infected with HCVcc were maintained in the presence (left panels) or absence (right panels) of IL28RA nAb (10 μg/mL). On Day 6 post-infection, cells were treated with 10 ng/mL of alfa or Lambda for 1 h, and then immunostaining was performed as described in the legend for Fig. 2 using antibodies directed against pSTAT1 (red) and the HCV-core antigen (green). Nuclei were counterstained with Hoechst dye (blue) as shown in the overlays. Arrows indicate examples of different HCV-pSTAT1 co-localization patterns in overlaid optical field. (TIF) [file pone.0121734.s005.tif]

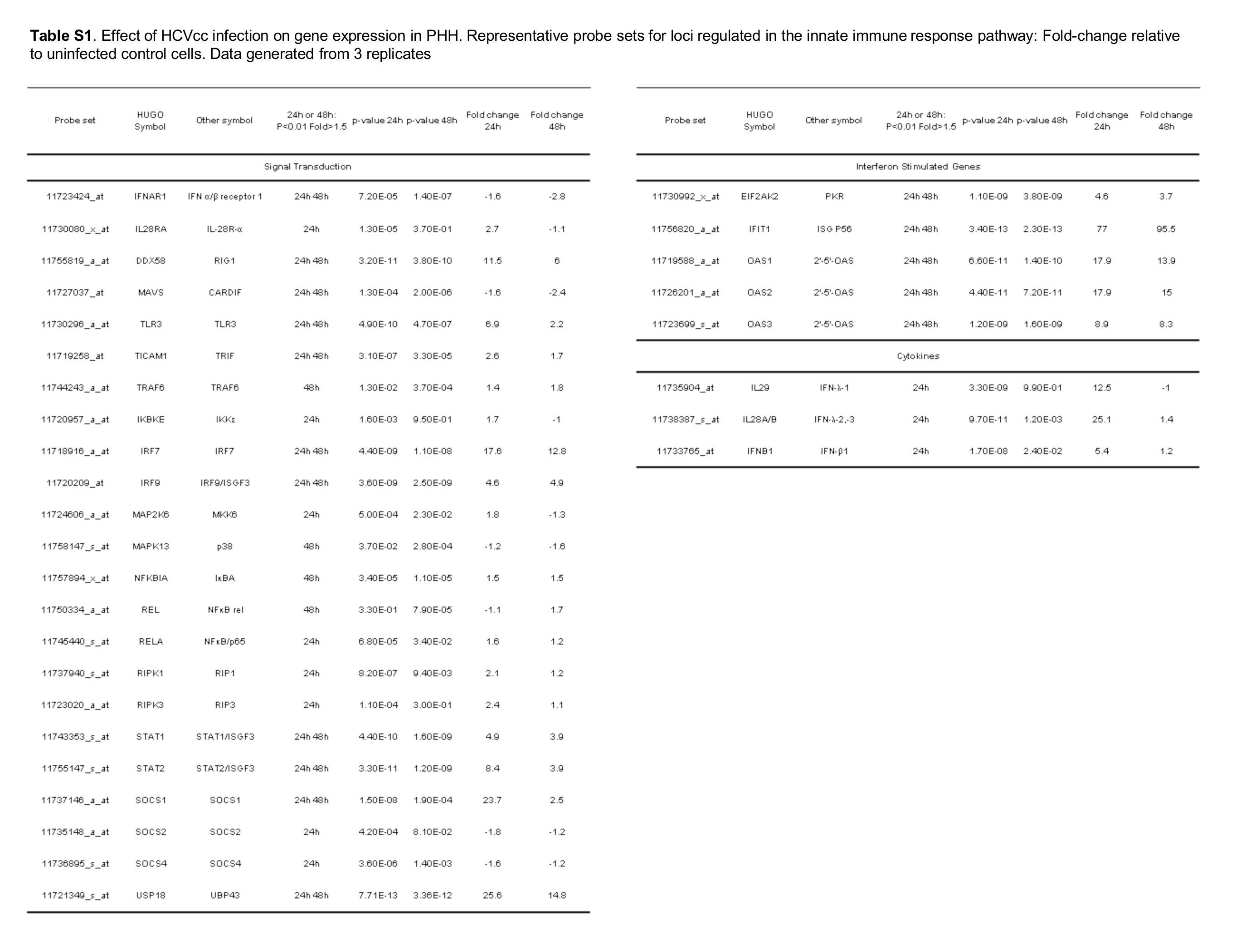

Supplement: S1 Table — Representative probe sets for loci regulated in the innate immune response pathway: Fold-change relative to uninfected control cells. Data generated from 3 replicates. (TIF) [file pone.0121734.s006.tif]

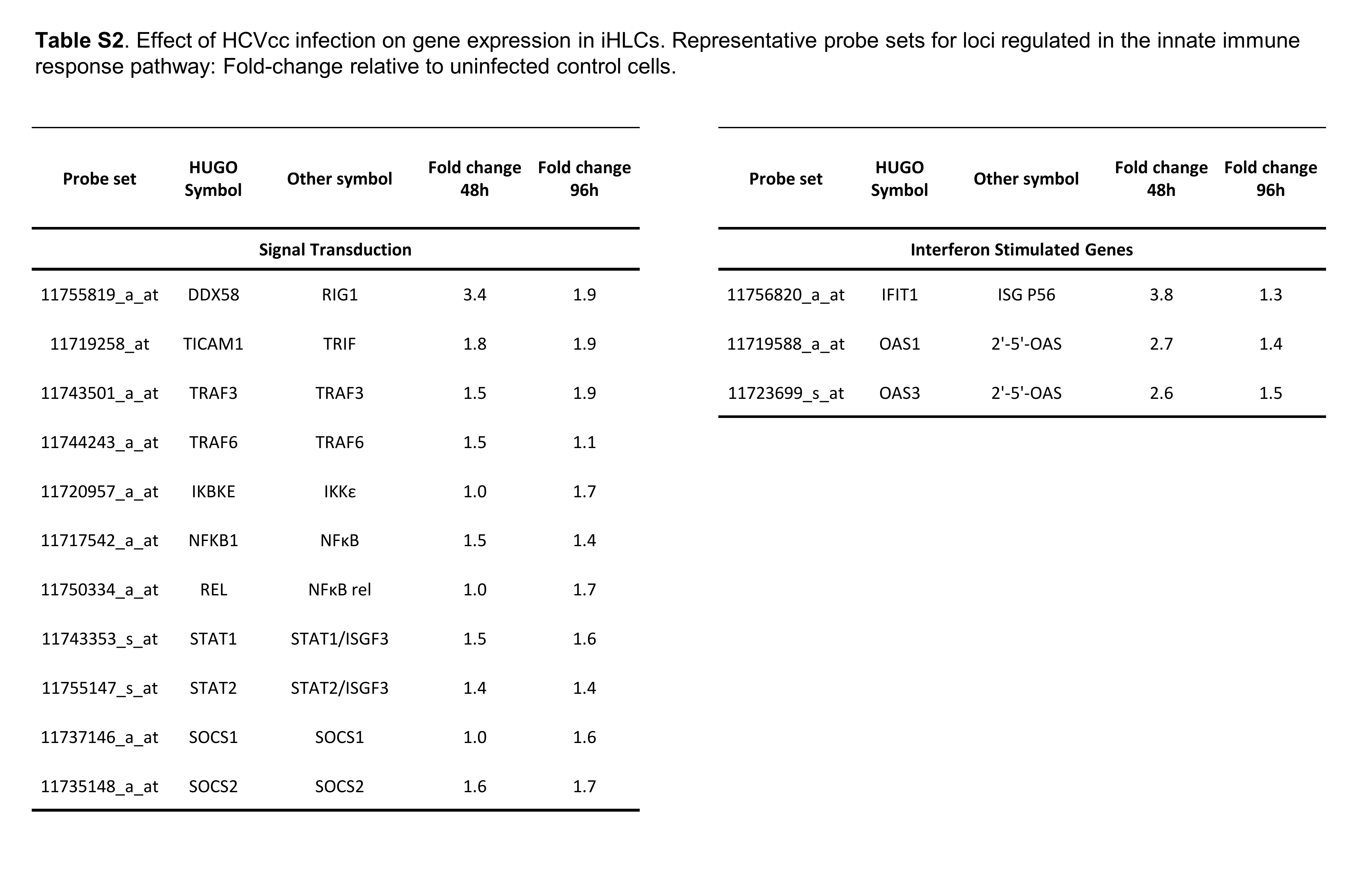

Supplement: S2 Table — Representative probe sets for loci regulated in the innate immune response pathway: Fold-change relative to uninfected control cells. (TIF) [file pone.0121734.s007.tif]

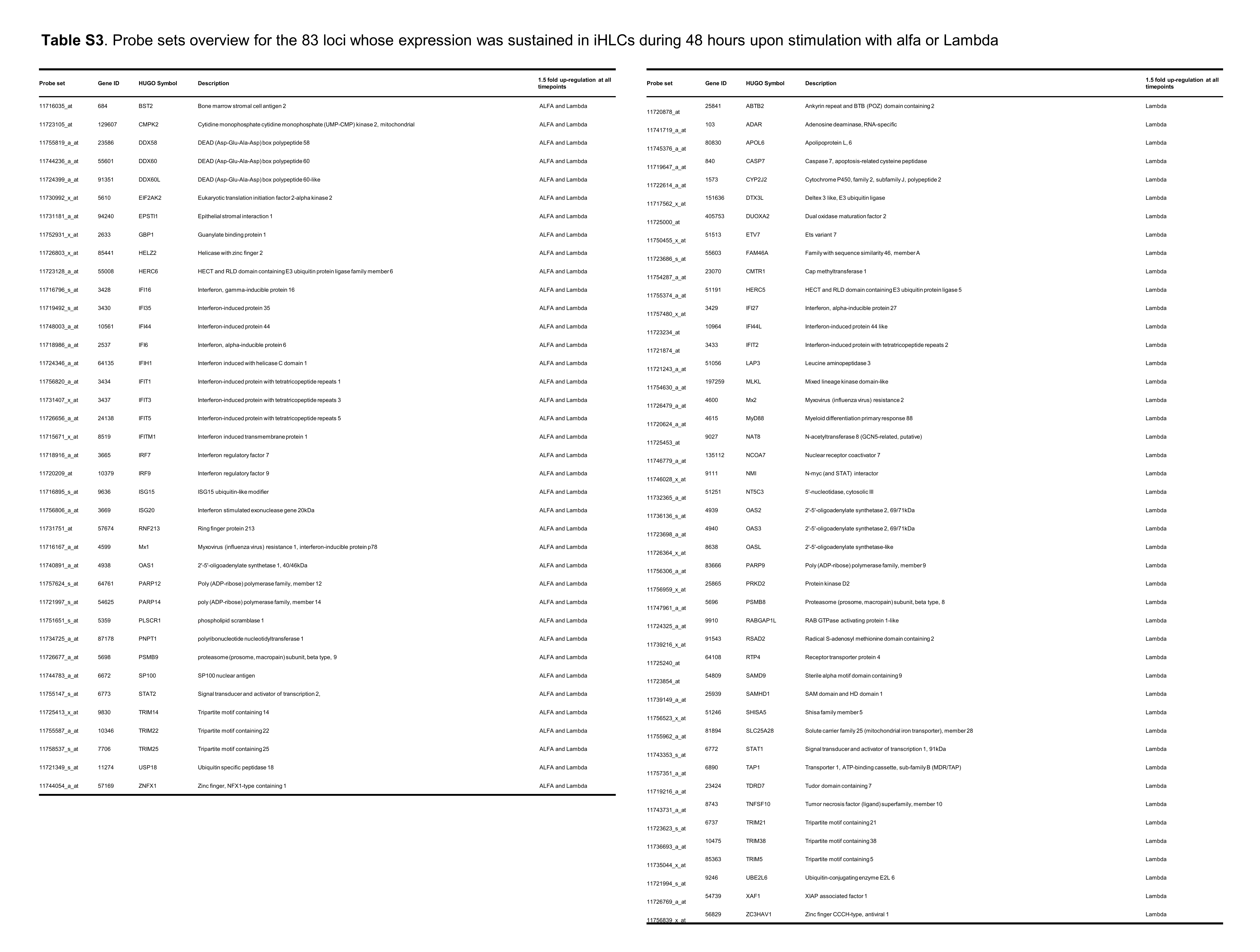

Supplement: S3 Table — (TIF) [file pone.0121734.s008.tif]
